# Supplementary material for: Clinically Relevant Dose of Pemafibrate, a Novel Selective Peroxisome Proliferator-Activated Receptor α Modulator (SPPARMα), Lowers Serum Triglyceride Levels by Targeting Hepatic PPARα in Mice
Source: Biomedicines. 2022 Jul 11;10(7):1667. doi: 10.3390/biomedicines10071667 (PMC9313206; doi:10.3390/biomedicines10071667)

Supporting information

Index of supplementary tables and figure:

Supplementary Table S1. Primer pairs used for qPCR analysis.

Supplementary Table S2. Primary antibodies used for western blot analysis.

Supplementary Table S3. Biochemical analysis of serum and liver.

Supplementary Figure S1. PEM does not affect FA/TG metabolism in eWAT.

Supplementary Figure S2. PEM does not affect FA/TG metabolism in BAT.

Supplementary Figure S3. Photomicrographs of hematoxylin and eosin-stained heart and kidney tissue.

Supplementary Figure S4. Clinically relevant dose of PEM activates hepatic PPARα without enhancing PPARβ or PPARγ.

Supplementary Figure S5. Full-length blots

Supplementary Table S1. Primer pairs used for qPCR analysis.

Gene	Accession #	Primer sequence (5'-3')
18S rRNA	NR_003278	F 5'-CACGGACAGGATTGACAGATTG-3'
		R 5'-CAGACAAATCGCTCCACCAA-3'
Acaa1	NM_130864	F 5'-TCTACGGTCAACAGACAGTGTTCA-3'
		R 5'-GGCCATGCCAATGTCATAAGA-3'
Acaca	NM_133360	F 5'-GGGCACAGACCGTGGTAGTT-3'
		R 5'-CAGGATCAGCTGGGATACTGAGT-3'
Acadl	NM_007381	F 5'-AGAAACATGGCGGCATTG-3'
		R 5'-CAATATCTGAGTGGAGGCTGAAG-3'
Acadm	NM_007382	F 5'-TGCTTTTGATAGAACCAGACCTACAGT-3'
		R 5'-CTTGGTGCTCCACTAGCAGCTT-3'
Acads	NM_007383	F 5'-CTCCACAGCTAACCTCATCTTTG-3'
		R 5'-GGGTTTGCATGGCTATTTTG-3'
Acadvl	NM_017366	F 5'-GCGTGTGCTCCGAGATATTC-3'
		R 5'-CCAGTGAGTTCCTTTCCTTTG-3'
Acox1	NM_015729	F 5'-TGGTATGGTGTCGTACTTGAATGAC-3'
		R 5'-AATTTCTACCAATCTGGCTGCAC-3'
Acsl1	NM_007981	F 5'-TCCTACGGCAGTGATCTGGTG-3'
		R 5'-GGTTGCCTGTAGTTCCACTTGTG-3'
Apob	NM_009693	F 5'-TCACCCCCGGGATCAAG-3'
		R 5'-TCCAAGGACACAGAGGGCTTT-3'
Cd36	NM_007643	F 5'-CCAAATGAAGATGAGCATAGGACAT-3'
		R 5'-GTTGACCTGCAGTCGTTTTGC-3'
Ces1d	NM_053200	F 5'-TGGTATTTGGTGTCCCATCA-3'
		R 5'-GCTTGGGCGATACTCAAACT-3'
Cpt1	NM_013495	F 5'-TGGCATCATCACTGGTGTGTT-3'
		R 5'-GGTCCGATTGATCTTTGCAATC-3'
Cpt2	NM_009949	F 5'-ATCGTACCCACCATGCACTAC-3'
		R 5'-CTGTCATTCAAGAGAGGCTTCTG-3'
Dgat1	NM_010046	F 5'-CTGCTACGACGAGTTCTTGAGA-3'
		R 5'-GATAGTAGGGACCATCCACTGTTG-3'
Dgat2	NM_026384	F 5'-GCTTCGCGAGTACCTGATGT-3'
		R 5'-CACCACGATGATGATAGCATTG-3'
Ehhadh	NM_023737	F 5'-CGATACTCTTCCCCCACTACCA-3'
		R 5'-CAGTTACCAACAACGACTCCAATC-3'
Fabp1	NM_017399	F 5'-GCAGAGCCAGGAGAACTTTGAG-3'
		R 5'-TTTGATTTTCTTCCCTTCATGCA-3'
Fasn	NM_007988	F 5'-ATCCTGGAACGAGAACACGATCT-3'
		R 5'-AGAGACGTGTCACTCCTGGACTT-3'

Hsl	NM_010719	F 5'-GAGCGCTGGAGGAGTGTTTT-3'<br>R 5'-TGATGCAGAGATTCCCACCTG-3'
Lipc	NM_008280	F 5'-ACGGGAAGAACAAGATTGGAAG-3'<br>R 5'-CGTTCCCTCAAACATAGGGC-3'
Lpl	NM_008509	F 5'-CGCTCCATTCATCTCTTCATT-3'<br>R 5'-GGCAGAGCCCTTTCTCAAAGG-3'
Mttp	NM_008642	F 5'-GAGCGGTCTGGATTTACAACG-3'<br>R 5'-GTAGGTAGTGACAGATGTGGCTTTTG-3'
Ppargc1a	NM_008904	F 5'-GACTCAGTGTCACCACCGAAATC-3'<br>R 5'-GACCTGTGTCGAGAAAAGGATCTT-3'
Pnpla2	NM_025802	F 5'-CGTGTTTCAGACGGAGAGAAC-3'<br>R 5'-TTGGAGGGTAGGAGGAATGAG-3'
Slc25a20	NM_020520	F 5'-GAGCCGAAACCCATCAGTCC-3'<br>R 5'-CAGTCGGACCTTGACCGTG-3'
Slc27a1	NM_011977	F 5'-ACCACCGGGCTTCCTAAGG-3'<br>R 5'-CTGTAGGAATGGTGGCCAAAG-3'
Ucp1	NM_009463	F 5'-AGGATGGTGAACCCGACAAC-3'<br>R 5'-GGCCTTCACCTTGGATCTGA-3'

F, forward sequence; R, reverse sequence.
Acaa1, acetyl-CoA acyltransferase 1
Acaca, acetyl-CoA carboxylase α
Acadl, long-chain acyl-CoA dehydrogenase
Acadm, medium-chain acyl-CoA dehydrogenase
Acads, short-chain acyl-CoA dehydrogenase
Acadvl, very long-chain acyl-CoA dehydrogenase
Acox1, acyl-CoA oxidase 1
Acsl1, long chain acyl-CoA synthase
Apob, apolipoprotein B
Cd36, fatty acid translocase
Ces1d, carboxylesterase 1d
Cpt1, carnitine palmitoyl transferase 1
Cpt2, carnitine palmitoyl transferase 2
Dgat1, diacylglycerol acyltransferase 1
Dgat2, diacylglycerol acyltransferase 2
Ehhadh, enoyl-Coenzyme A, hydratase/3-hydroxyacyl Coenzyme A dehydrogenase
Fabp1, fatty acid binding protein 1
Fasn, fatty acid synthase
Hsl, hormone-sensitive lipase
Lipc, hepatic lipase
Lpl, lipoprotein lipase
Mttp, microsomal triglyceride transfer protein

Ppargc1a, peroxisome proliferator-activated receptor gamma coactivator 1 alpha
Pnpla2, patatin-like phospholipase domain containing 2
Slc25a20, mitochondrial carnitine/acylcarnitine translocase
Slc27a1, fatty acid transport protein 1
Ucp1, uncoupling protein 1

Supplementary Table S2. Primary antibodies used for western blot analysis.

Protein name	Abbreviation	Manufacturer	Catalog #	Dilution
4-hydroxy-nonenal modified protein	4HNE	JalCA (Shizuoka, Japan)	#MHN-020P	1:100
Acetyl-CoA carboxylase alpha	ACCα	Santa Cruz Biotechnology (Dallas, TX)	#sc-30212	1:200
Actin, cytoplasmic 1	ACTB	Abcam (Cambridge, MA)	#ab8227	1:1000
Cluster of differentiation 36	CD36	Biotechnology (Dallas, TX)	#sc-9154	1:200
Fatty acid synthase	FAS	Biotechnology (Dallas, TX)	#sc-16147	1:200
Histone H1	Histone H1	Santa Cruz Biotechnology (Dallas, TX)	#sc-10806	1:200
Microsomal TG transfer protein	MTP	Santa Cruz Biotechnology (Dallas, TX)	#sc-33116	1:200
Peroxisome proliferator-activated receptor α	PPARα	Santa Cruz Biotechnology (Dallas, TX)	#sc-9000	1:200

Other antibodies used were described previously (Aoyama et al., J Biol Chem 1998).

Supplementary Table S3. Biochemical analysis of serum and liver.

Serum biochemical characteristics

**Item**	**Control group** (n=8)	**PEM-0.1 group** (n=10)	**PEM-0.3 group** (n=9)
Glucose (mg/dL)	205.8 ± 55.90	212.93 ± 75.06	174.96 ± 35.61
Insulin (ng/dL)	0.37 ± 0.13	0.30 ± 0.12	0.19 ± 0.14
HMW adiponectin (mg/dL)	129.86 ± 93.27	107.36 ± 67.91	104.53 ± 58.63

Liver biochemical characteristics

**Item**	**Control group** (n=8)	**PEM-0.1 group** (n=10)	**PEM-0.3 group** (n=9)
PL (mg/g liver)	48.03 ± 10.85	43.19 ± 9.48	47.23 ± 14.19

Values are expressed as the mean ± SD.

Supplementary Figure S1. PEM does not affect FA/TG metabolism in eWAT. (**A**) Representative photomicrographs of hematoxylin and eosin-stained eWAT sections. Scale bar= 50 μm. (**B-D**) Hepatic mRNA levels of genes related to FA uptake (*Cd36*), FA synthesis (*Fasn* and *Acaca*), TG synthesis (*Dgat1* and *Dgat2*), TG decomposition (*Lpl, Ces1b*, *Hsl* and *Pnpla2*), and fat burning (*Ppargc1a*) were quantified by qPCR, normalized to that of 18s ribosomal RNA levels, and expressed as values relative to those of control mice. Data are expressed as the mean ± SD. Con, vehicle-treated mice; PEM-0.1, pemafibrate-treated mice at a clinically relevant dose (0.1 mg/kg/day); PEM-0.3, pemafibrate-treated mice at a relatively high dose (0.3 mg/kg/day).

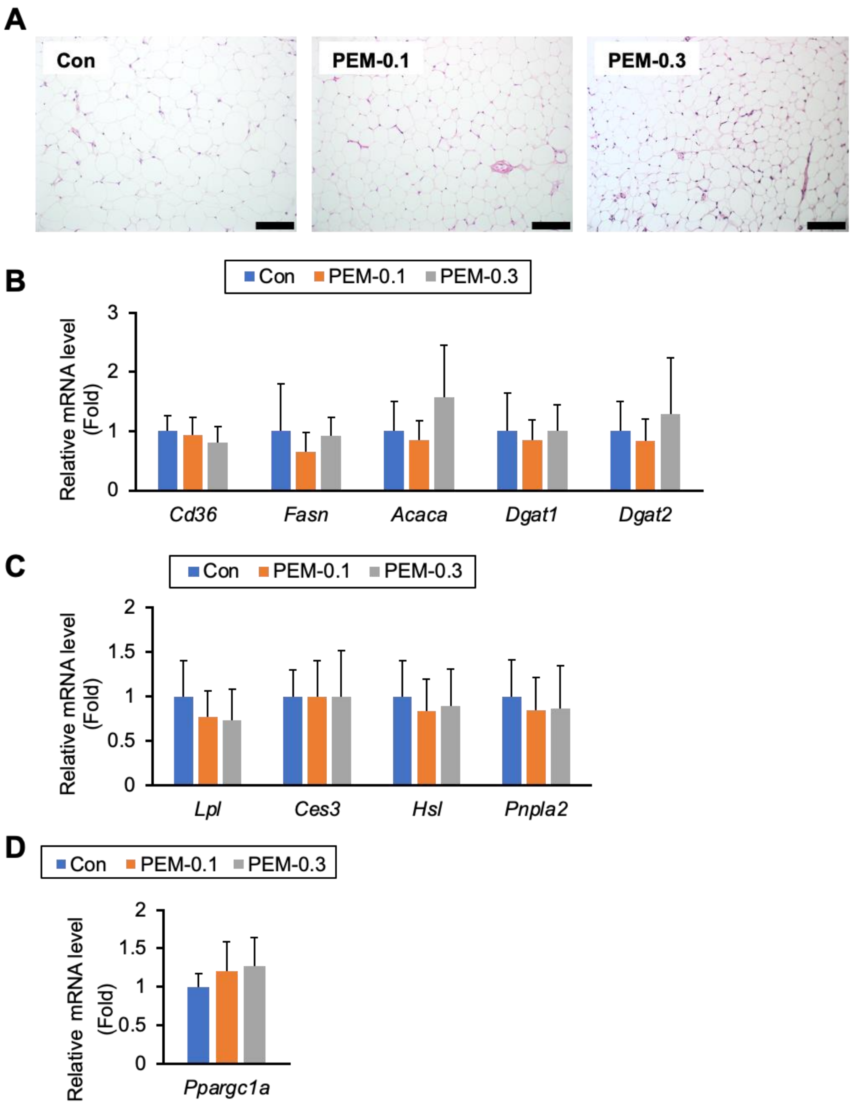

Supplementary Figure S2. PEM does not affect FA/TG metabolism in BAT. (**A**) Representative photomicrographs of hematoxylin and eosin-stained BAT sections. Scale bar= 50 µm. (**B-D**) Hepatic mRNA levels of genes related to FA uptake (*Cd36*), intracellular FA transport (*Slc27a1*), TG decomposition (*Lpl* and *Hsl*), and fat burning (*Ppargc1a* and *Ucp1*) were quantified by qPCR, normalized to that of 18s ribosomal RNA levels, and expressed as values relative to those of control mice. Data are expressed as the mean ± SD. Con, vehicle-treated mice; PEM-0.1, pemafibrate-treated mice at a clinically relevant dose (0.1 mg/kg/day); PEM-0.3, pemafibrate-treated mice at a relatively high dose (0.3 mg/kg/day).

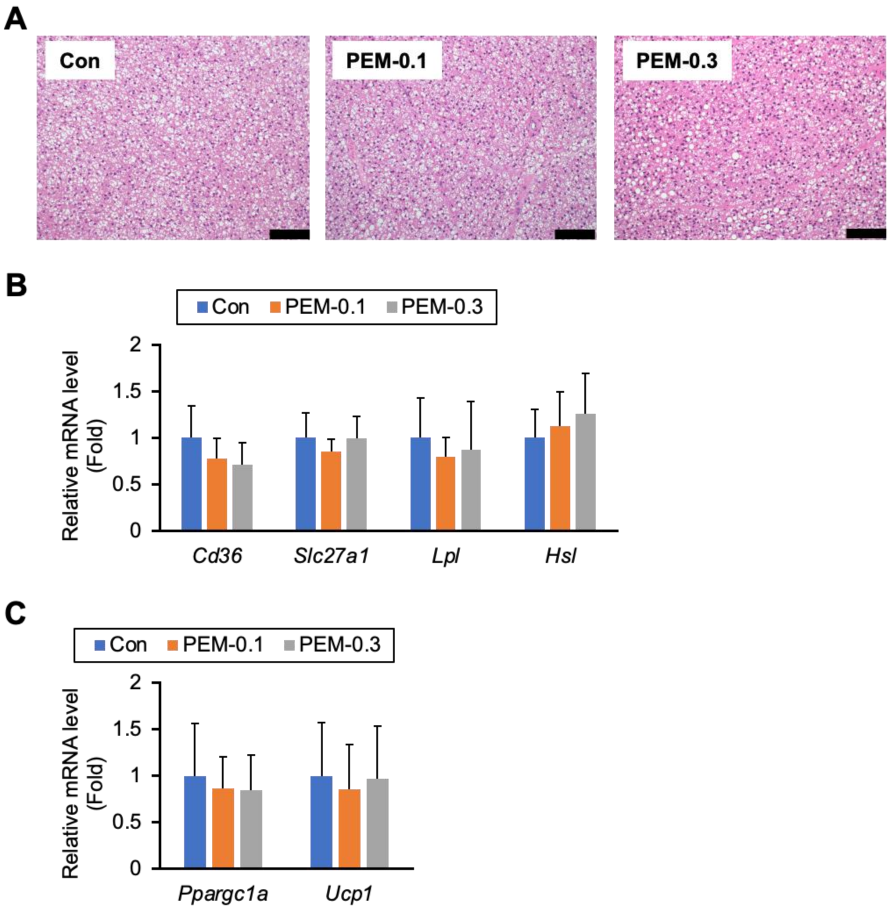

Supplementary Figure S3. Representative photomicrographs of hematoxylin and eosin-stained heart (A) and kidney (B) tissue.

Scale bar= 50 μm. Con, vehicle-treated mice; PEM-0.1, pemafibrate-treated mice at a clinically relevant dose (0.1 mg/kg/day); PEM-0.3, pemafibrate-treated mice at a relatively high dose (0.3 mg/kg/day).

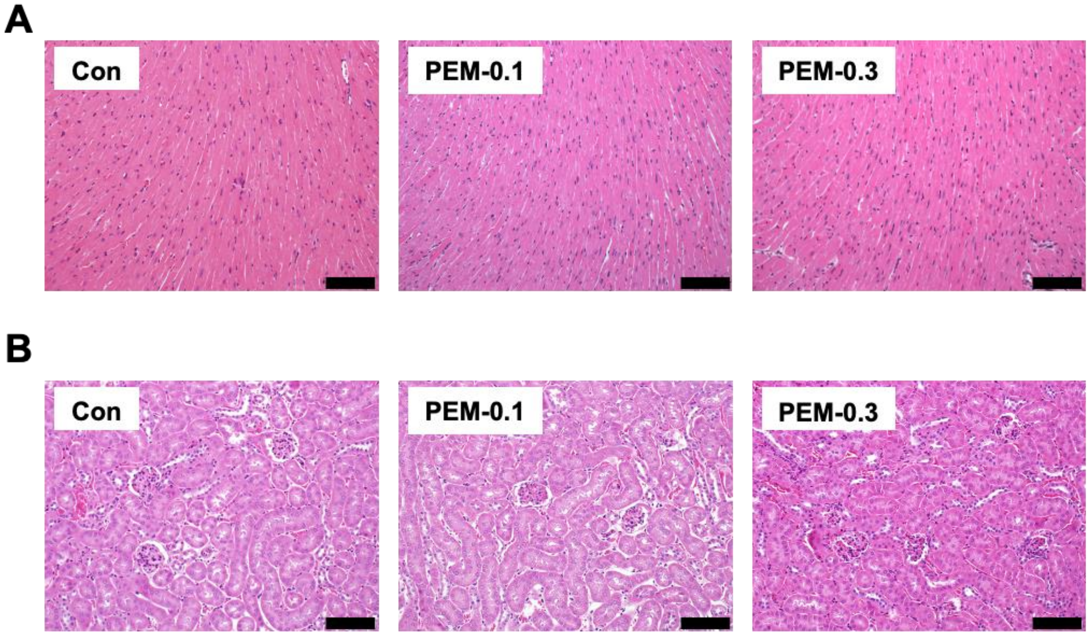

Supplementary Figure S4. Clinically relevant dose of PEM activates hepatic PPARα without enhancing PPARβ or PPARγ.

Immunoblot analysis of PPARβ and PPARγ. The band of histone H1 was used as a loading control. Data are expressed as the mean ± SD. Con, vehicle-treated mice; PEM-0.1, pemafibrate-treated mice at a clinically relevant dose (0.1 mg/kg/day); PEM-0.3, pemafibrate-treated mice at a relatively high dose (0.3 mg/kg/day).

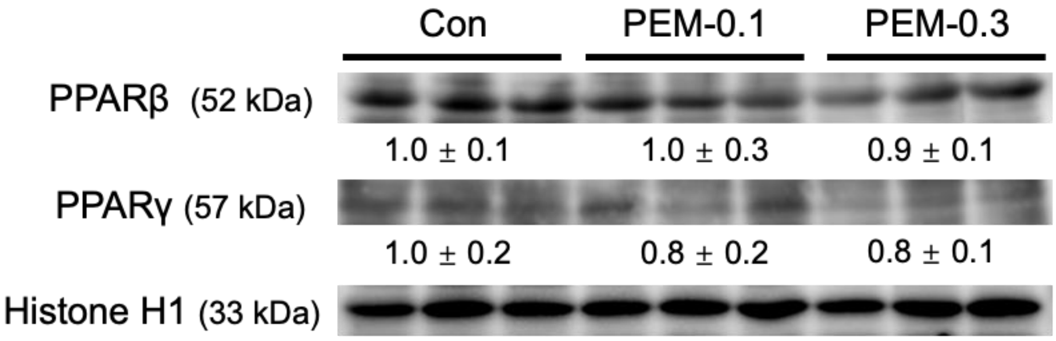

Supplementary Figure S5. Full-length blots.

The protein bands inside the black dotted lines are used in this paper.

Figure 3

B

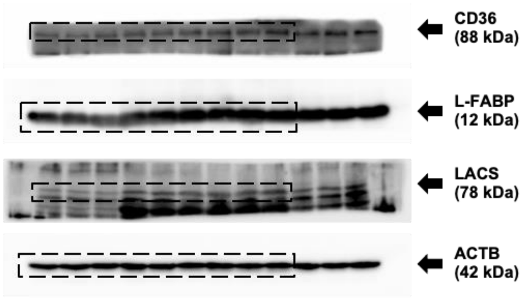

Figure 4

B

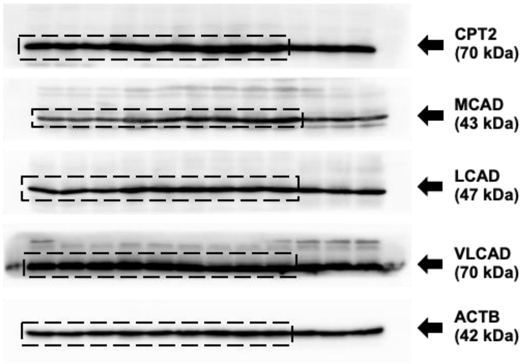

Figure 5

B

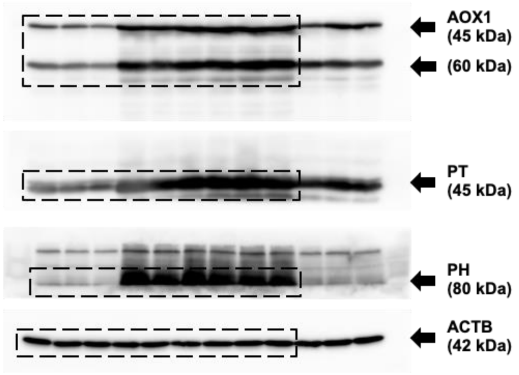

Figure 6

B

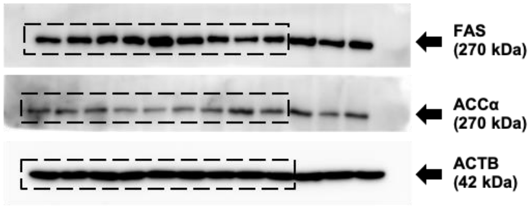

D

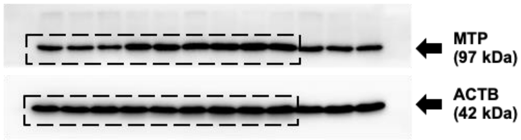

Figure 8

A

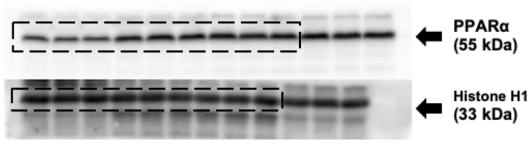

B

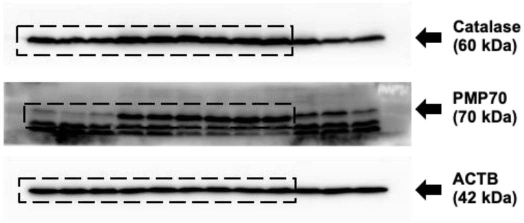

C

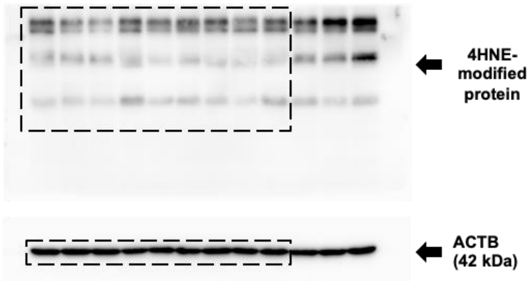

Figure S4

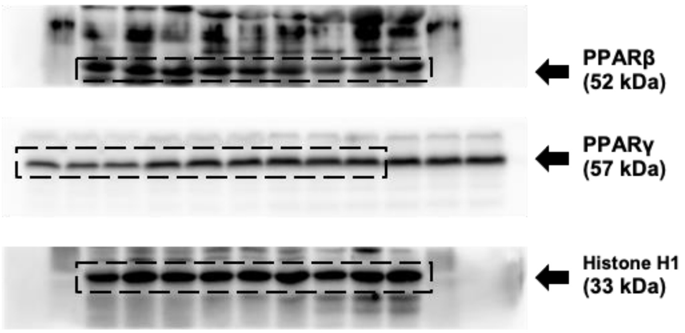

Supplement: Supplementary file 1 [file biomedicines-10-01667-s001.zip › biomedicines-1756236-supplementary.pdf]
